# Micro- and Macrostructured PLGA/Gelatin Scaffolds Promote Early Cardiogenic Commitment of Human Mesenchymal Stem Cells In Vitro

**DOI:** 10.1155/2016/7176154

**Published:** 2016-10-16

**Authors:** Caterina Cristallini, Elisa Cibrario Rocchietti, Mariacristina Gagliardi, Leonardo Mortati, Silvia Saviozzi, Elena Bellotti, Valentina Turinetto, Maria Paola Sassi, Niccoletta Barbani, Claudia Giachino

**Affiliations:** ^1^Institute for Chemical-Physical Processes, IPCF C.N.R., UOS Pisa, 56122 Pisa, Italy; ^2^Department of Clinical and Biological Sciences, University of Turin, Orbassano, 10043 Turin, Italy; ^3^Center for Micro-BioRobotics @SSSA, Istituto Italiano di Tecnologia, Viale Rinaldo Piaggio 34, 56025 Pontedera, Italy; ^4^National Institute of Research in Metrology, INRIM, 10135 Turin, Italy; ^5^Department of Civil and Industrial Engineering, University of Pisa, 56122 Pisa, Italy

## Abstract

The biomaterial scaffold plays a key role in most tissue engineering strategies. Its surface properties, micropatterning, degradation, and mechanical features affect not only the generation of the tissue construct in vitro, but also its in vivo functionality. The area of myocardial tissue engineering still faces significant difficulties and challenges in the design of bioactive scaffolds, which allow composition variation to accommodate divergence in the evolving myocardial structure. Here we aimed at verifying if a microstructured bioartificial scaffold alone can provoke an effect on stem cell behavior. To this purpose, we fabricated microstructured bioartificial polymeric constructs made of PLGA/gelatin mimicking anisotropic structure and mechanical properties of the myocardium. We found that PLGA/gelatin scaffolds promoted adhesion, elongation, ordered disposition, and early myocardial commitment of human mesenchymal stem cells suggesting that these constructs are able to crosstalk with stem cells in a precise and controlled manner. At the same time, the biomaterial degradation kinetics renders the PLGA/gelatin constructs very attractive for myocardial regeneration approaches.

## 1. Introduction

In the paradigm of tissue engineering, a three-dimensional platform known as scaffold is essential for cell proliferation, growth and differentiation, and the final generation of a functional tissue [1, 2].

Generally, scaffolds in tissue engineering have to satisfy simultaneously a wide series of requirements that render it difficult to obtain a functional structure suitable for clinical use.

If the tissue to regenerate is the myocardium, the difficulty in controlling the engineering of a scaffold mimicking the complex tissue organization is even greater. The structural components of cardiac tissue are arranged both in micro- (cardiac muscle fibers) and in nanoscale (filaments, sarcomeres); however, it is not well clear which are the most important topographical initial stimuli to activate the complex process that can lead to in vivo engineering of cardiac tissue.

Many efforts were devoted to develop a scaffold able to reproduce at nano-, micro-, and mesoscale levels the biochemical, mechanical, and structural characteristics of cardiac extracellular matrix (ECM). In this regard, Kim et al. demonstrated that the engineered tissue structure and function are highly sensitive to changes of topographic features at nanoscale level [3]. It was observed that the nanofabrication of hydrogels based on PEG diacrylate can regulate the alignment of neonatal rat ventricular myocytes and guide structural and functional cardiac anisotropy [3–5]. Moreover, a variety of substrates with micron patterns have been studied to direct cardiomyocytes into anisotropic organized cardiac tissue constructs using different microfabrication techniques [6–8]. Engelmayr Jr. et al. showed the formation of grafts with preferentially aligned neonatal rat heart cells and mechanical properties more closely resembling adult rat right ventricular myocardium [9].

Besides the use of differentiated heart cells, most relevant for regenerative approaches is the influence of engineered scaffolds on stem cells. Human mesenchymal stem cells (hMSCs) can be readily harvested from bone marrow through aspiration. Due to their high self-renewal capacity and excellent differentiation potential in vitro, hMSCs are ideally suited for regenerative medicine [10]. The complex molecular and functional interactions of MSCs with their environment are widely studied but not completely understood. The characteristics of the physical microenvironment that have been already shown to modulate MSC fate in different tissues include ECM stiffness [11], interactions with nanoscale features [12], anisotropy [13], and the micropattern [14, 15]. Other physical features might turn out to be critical in influencing stem cells for cardiac tissue engineering approaches including the scaffold degradation characteristics. Indeed, in view of an in vivo approach, to establish the best scaffold from a degradation rate point of view to match the regeneration/healing process is of fundamental importance.

Polymeric biomaterials produced using PLGA offer important advantages including biocompatibility, biodegradation that can be modulated by varying PLA and PGA fraction content, and, once functionalized with drugs, the possibility to accurately control the release kinetics to reach adequate administration [16]. In addition, PLGA has been approved for several biomedical applications in human and used as scaffold materials in tissue engineering [17]. Even if the use in the cardiovascular field is not largely explored, however PLGA scaffolds, mainly in form of nanofibers obtained by electrospinning, were extensively studied in orthopedic applications [18–22]. PLGA constructs functionalized with TGF-*β* were shown to influence the growth and differentiation behavior of MSCs into osteoblasts and chondrocytes [23, 24]. Moreover, PLGA scaffolds incorporating SDF1-*α* were studied to evaluate stem cell recruitment and inflammation response to improve critical tissue response upon scaffold implant [25]. Another study demonstrated that nano- and microstructured electrospun non-woven PLGA-based scaffolds provided both flexibility and guidance for cardiac myocytes growth [26] and recently an omentum-wrapped PLGA patch seeded with rat MSCs was grafted onto the epicardium of the infarction area demonstrating beneficial effects on ventricular remodeling and cardiac function [27]. Gelatin is one of the most used natural materials to recreate the native myocardial microenvironment [28]. In the past, pure gelatin scaffolds have been applied for in vitro and in vivo cardiac tissue regeneration [29]. Recently, micromolded gelatin hydrogels have demonstrated to support long-term culture of engineered rat and human cardiac tissues [30]. Moreover, a phase I clinical trial was performed using a combination of gelatin hydrogel embedded with human cardiac-derived stem cells and basic fibroblast growth factor (bFGF), even if a deepened follow-up is necessary to evaluate treatment efficacy [31]. In addition, a recent study carried out on gelatin-based hydrogels incorporating gold nanorods suggested their possible use as cardiac patches with superior electrical and mechanical properties [32].

In a previous paper we demonstrated the capability of scaffolds based on poly(3-hydroxybutyrate-co-3-hydroxyvalerate)/gelatin (PHBHV/gelatin) to induce stem cell commitment to a cardiac phenotype thanks to an optimal synergy between physico-chemical, mechanical, and structural characteristics [33].

In this paper we evaluated hMSC behavior on a scaffold with analogous micropatterning and bioartificial features but different for what concerns the synthetic polymer used, poly(DL-lactide-co-glycolide) (PLGA), and blending procedure. In addition, a modification at the macroscale level by producing a bilayered microstructured scaffold was setup, in order to increase the thickness of the patch, while maintaining the surface microstructure, to create a more robust and adaptable construct to the extent of damaged tissue and to use it in the future as reservoir for the release of specific drugs. PLGA/gelatin scaffolds were prepared and characterized under many aspects including anisotropy, molecular interaction, and degradation process in order to better investigate their capability to mediate the cardioinductivity in view of the realization of an efficient cardiac patch.

Here we show that microstructured PLGA/gelatin scaffolds have a rapid degradation kinetics, while maintaining the biomechanical stimuli induced by micropatterning, and provide a suitable matrix for hMSC adhesion, organized growth, and early cardiac commitment.

## 2. Materials and Methods

### 2.1. Preparation and Physicochemical/Mechanical Characterization of PLGA/Gelatin Scaffolds

#### 2.1.1. Reagents

Poly(DL-lactide-co-glycolide) (Sigma Aldrich, lactide : glycolide (50 : 50), M.W. 40–75 kDa), gelatin from porcine skin (Sigma Aldrich), Milli-Q water (Millipore), dichloromethane (DCM), and acetone (ACT) tetrahydrofuran (THF) (Carlo Erba Reagenti, Italy).

#### 2.1.2. Preparation of Bioartificial Blends

Bioartificial blends based on PLGA and gelatin were obtained by means of controlled solvent casting starting from water/ACT/DCM ternary blends. In detail, a predefined volume of gelatin is added to a solution of PLGA (10%) in DCM and ACT to obtain different composition PLGA/gelatin (90/10, 80/20, 75/25, 70/30 w/w). Successively the composition PLGA/gelatin 70/30 will be selected, mainly on the basis of mechanical properties and specifically lower stiffness (Figure 2(c)).

#### 2.1.3. Preparation of ECM-Like Porous Bioartificial Scaffolds

The bioartificial blends were spread onto silicon mould obtained by soft lithography technique as previously reported [33]; the mould presents on the surface a micropatterning having a predefined geometry mimicking the myocardium anatomic microstructure [34]. Each layer is designed to possess channels with dimensions of 500 *μ*m × 100 *μ*m and a depth of 60 *μ*m, separated by an array of reliefs of 70 *μ*m and 30 *μ*m alternatively and cross reliefs of 30 *μ*m. After spreading the bioartificial blend was undergone to a slow and controlled casting and finally the material was gently detached by the mould by obtaining a microstructured single layer. For the preparation of bilayer, an aliquot of gelatin solution was homogenously deposited on a single layer from the side without any cavities and reliefs. A second microstructured layer was assembled leaving the micropatterning to the free side. Finally, the layers were fixed by a bland and rapid die-casting. For the die-casting, metal moulds at rectangular profiles were prepared having the same size of the scaffold surface. The moulds were heated at a maximum temperature of 50°C and positioned at the edges of the bilayer.

#### 2.1.4. Scaffold Characterization

Morphological analysis was carried out onto Au sputtered samples by scanning electron microscopy (SEM, Jeol JSM 5600, Japan). Chemical analysis was carried out by FT-IR Chemical Imaging (Perkin Elmer Spotlight 300, USA): attenuated total reflectance (ATR) sampling technique on fragments of scaffolds was performed, the use of the Chemical Imaging allowed evaluating the distribution of components in the materials, obtaining chemical and correlation maps to visualize their distribution, spectral images were acquired in transmission and *μ*ATR mode (spectral resolution was 4 cm^−1^, spatial resolution was 100 × 100 *μ*m) using the infrared imaging system Spotlight 300 (Perkin Elmer). Spectra were collected by touching the ATR objective on the sample and collecting the spectrum generated from the surface layer of the sample. The Spotlight software used for acquisition was also used to preprocess the spectra; dynamic mechanical analysis was performed by DMA (DMA8000, Perkin Elmer, USA), using both tensile tests to evaluate stress-strain curve, elastic modulus and yield strength, and strain scan analysis to evaluate storage modulus (*E*′) and loss modulus (*E* ^″^) of the not microstructured materials in dry condition, for microstructured scaffolds strain scan analysis was carried out in dry and wet conditions at 37°C applying the strain in parallel and perpendicular directions with respect to the microstructure alignment. Preliminary degradation tests were performed on different scaffold composition in MilliQ water at 37°C to evaluate degradation kinetics at early time. Then, on selected scaffolds in form of bilayer, degradation analysis was performed up to six months in three different media: MilliQ water, Phosphate Buffered Saline (PBS, pH 7.4, Sigma), and Dulbecco's Modified Eagle Medium (DMEM, Life Technologies, USA). Degradation samples underwent SEM, FT-IR, and gel permeation chromatography (GPC, Perkin Elmer, USA) analyses; SEM analysis allowed evaluating how scaffold morphology changed after degradation; FT-IR evaluated the variation of chemical composition of samples; finally, GPC quantified the molecular weight lowering and the variation of polydispersity index of PLGA and PLGA/gelatin matrices obtaining degradation kinetic profiles. For GPC analysis, a Perkin Elmer pump, equipped by a ResiPore (Agilent Technologies, USA) column, UV and RI detectors, were used; internal mobile phase was composed of THF (1 mL/min); analysis was carried out at room temperature; results were obtained based on a calibration curve obtained with polystyrene narrow standards. Moreover, pH of degradation media was monitored during time using pH-meter (CRISON, basic 20).

### 2.2. Cell Seeding and Culture

hMSCs were obtained from Lonza Group (Walkersville, MD). Cells were tested for purity by flow cytometry using a panel of cell surface markers: CD105, CD44, CD29, CD90, CD166, CD34, CD45, and HLA-DR (all from Miltenyi, Germany) as previously described [33]. hMSC differentiation potential into osteocyte or adipocyte lineages was assessed as described [35]. hMSCs were used in experiments at passages 3 to 7. Depending on the experiments and the scaffold layer composition, between 5 × 10^3^ and 20 × 10^3^ cells were suspended in 50 *μ*L of DMEM (Life Technologies) supplemented with 10% fetal bovine serum (FBS, Euroclone, Italy), 2 mM L-glutamine, 1% kanamycin, 1% sodium pyruvate, 1% nonessential amino acids, 0.1% *β*-mercaptoethanol (all from Gibco, Gaithersburg, MD, USA) and seeded in drops on the surface of the PLGA/gelatin scaffolds placed in 24-well plates. After 3 hr necessary for cell adhesion, 500 *μ*L of complete medium was added to cover the scaffolds and samples were kept in an atmosphere of 5% CO_2_, 95% air at 37°C in a humidified incubator. Cells seeded on the scaffolds were maintained in culture for up to two weeks and medium was replaced every 3 days. For the 2-D control condition, 4 × 10^3^ cells/cm^2^ were seeded in 24-well plates and harvested at 80% confluence.

### 2.3. Adhesion and Proliferation Assays

Cell adhesion was evaluated after 24 hr of culture using the metabolic assay CellTiter-Blue (Promega, USA) as previously described [33].

### 2.4. Cell Morphology and Alignment Analyses

Cell morphology, cytoskeletal organization, and scaffold colonization were analysed by Immunofluorescence. Cellularized scaffolds were fixed with 4% paraformaldheyde (PFA, Sigma-Aldrich) for 30 min, permeabilized with 0.1% Triton X-100 (Sigma-Aldrich) for 15 min and blocked with 6% bovine serum albumin (BSA, Sigma-Aldrich) and 2.5% normal goat serum (NGS, Sigma-Aldrich) for 1 hr, and stained with red phalloidin 1 : 200 (Sigma-Aldrich) for 30 min and Hoechst 33342 1 : 10000 (Sigma-Aldrich) for 10 min, respectively. After mounting with Mowiol (Calbiochem, USA), cellularized scaffolds were analyzed by both confocal microscopy (TCS-SPE, Leica Microsystem, Germany) and Nonlinear Optics Multimodal Coherent Anti-Stokes Raman Scattering (CARS)/Two-Photon Excitation Fluorescence (TPEF) microscopy (see below).

Cell alignment and elongation were evaluated at 2, 4, 8, and 15 days of culture with 2 *μ*m Calcein-AM (Sigma-Aldrich). Cellularized scaffolds were stained with 2 *μ*M Calcein-AM staining and confocal microscopy analysis (Carl Zeiss Laser Scanning System LSM 510, Germany). 3D reconstructions were obtained with Imaris 7.6.1 (Bitplane Scientific Solution, Switzerland). hMSC mean cell length was analyzed using ImageJ® ROI measure tool (Rasband, W.S., ImageJ, U.S. National Institutes of Health, USA, http://rsb.info.nih.gov/ij/, 1997–2012). 2D control culture measurements refer to the mean cell length evaluated on confocal images of hMSCs cultured for 3 days on 24-well plates, a time when they reach the maximum elongation.

Employing the multimodal CARS/TPEF microscope realized as described in a previous paper [36], the PLGA/gelatin scaffold was 3D imaged simultaneously with the hMSCs using CARS and TPEF techniques, respectively. CARS signal was measured in forward detection, in a spectral range between 716 nm and 770 nm, while TPEF signal in epidetection in a spectral range between 510 nm and 570 nm. The lasers power onto the biological sample was regulated by a neutral density variable filter wheel. A water immersion objective lens (Olympus LUMPLFLN 60XW NA = 1, W.D. = 2 mm) was used to focus the excitation beams on the samples and for collecting TPEF signal, while an air objective lens (Olympus UPLSAPO 20x objective NA = 0.75) was used to collect CARS signal. PLGA/gelatin scaffold structures were imaged using CARS, looking for the C-H stretching Raman modes around 2940 cm^−1^, tuning the OPO pump and the Stokes beams to 920.5 nm and 1262.1 nm, respectively, and generating the CARS signal at a wavelength around 724.4 nm. The same pump beam at 920.5 nm was used to excite Calcein-AM for TPEF cell imaging. The sample (scaffold + cells) was simultaneously imaged by CARS and TPEF, collecting 41 images at different* Z* positions spaced by 2.5 *μ*m for a total thickness of 100 *μ*m. Each image was six times Kalman averaged. A viewer plugins on FIJI [37] allowed the 3D sample images reconstructions.

### 2.5. RNA Extraction, cDNA Synthesis, and Quantitative Reverse Transcriptase PCR (qPCR)

Cellularized scaffolds were dissolved in TRIzol reagent (Life Technologies) and total RNA (totRNA) was extracted according to the manufacturer's instructions. Genomic DNA contaminations were removed by DnaseI treatment (Ambion, USA). One microgram of totRNA was retrotranscribed with random hexamer primers using High Capacity Reverse Transcription Kit (Applied Biosystems, USA) in accordance with the manufacturer's suggestions. Expression levels of target genes were evaluated with SYBER green technology on an ABI PRISM 7500 Fast Real-Time PCR system (Applied Biosystems) using 25 ng of cDNA as template and 150 *μ*M of each primer (listed in Table 1).

Melting curve analysis was performed for all amplicons. For each target gene, fold change in expression levels between cellularized scaffolds and 2D control culture was evaluated with the 2^−ΔΔCt^ method using Pol2r as reference gene and matched 2D control culture as calibrators.

### 2.6. Cardiomyogenesis Immunofluorescence Analysis

After 15 days of culture both 2D control cells and cells seeded on the scaffolds were detached using 0.25% trypsin-1 mM EDTA (Life Technologies) and cytospinned at 250 rpm for 6 min. Cells were then fixed with 4% PFA, permeabilized with 0.1% Triton X-100, and blocked in 10% FBS for 1 hr, incubated with anti-c-kit (1 : 200) and anti-GATA-4 (1 : 100) antibodies (both from Santa-Cruz, USA) followed by an anti-rabbit secondary antibody (1 : 1000) (Sigma-Aldrich); cell nuclei were stained with Hoechst 33342 (Sigma-Aldrich) 1 : 1000. Coverslips were mounted with Mowiol and analyzed by confocal microscopy (TCS-SPE, Leica Microsystems).

### 2.7. Statistical Analysis

All results were expressed as mean ± standard error of the mean (S.E.M.). Statistically significant differences between any two groups were determined using the paired Student's* t*-test and differences with *p* ≤ 0.05 were regarded as statistically significant.

## 3. Results

### 3.1. PLGA/Gelatin Myocardial Scaffold Preparation and Characterization

#### 3.1.1. Nonmicrostructured PLGA/Gelatin Materials

**Figure 1:**
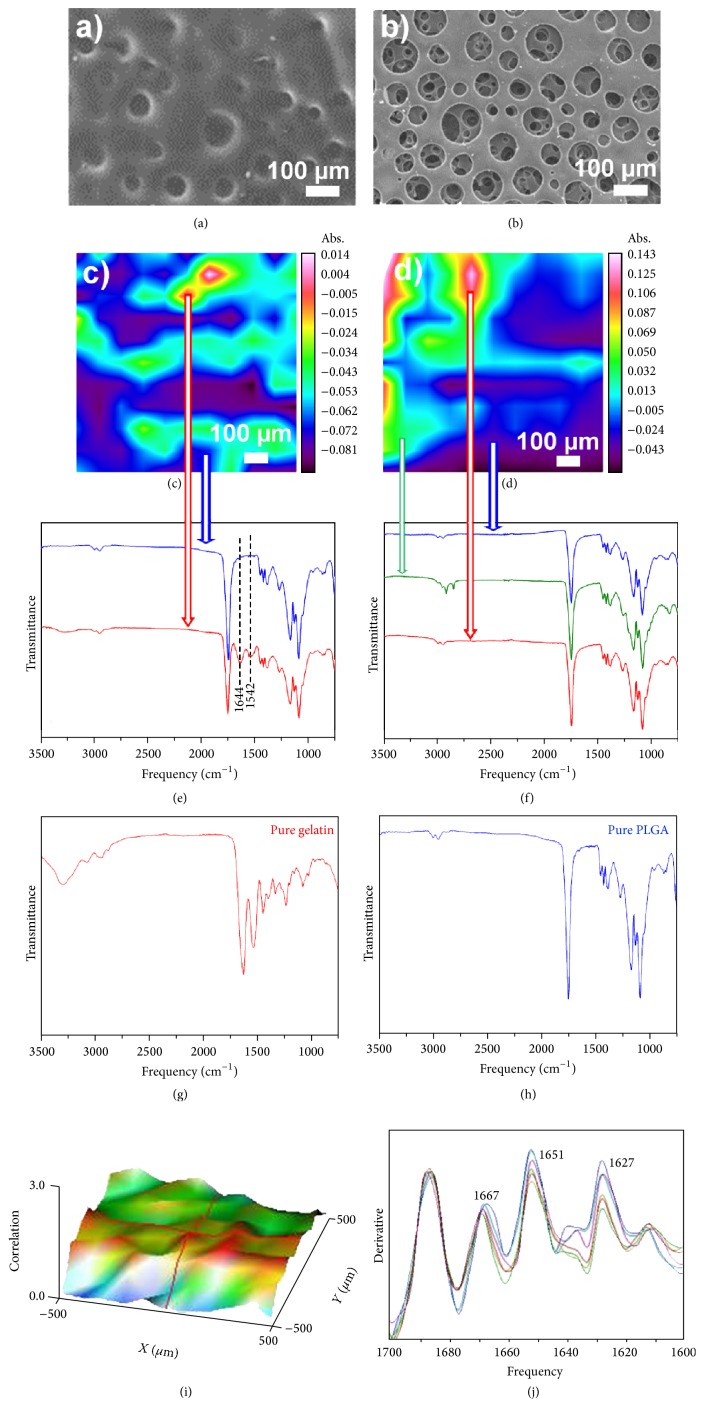
SEM images and Chemical Imaging analysis of nonmicrostructured PLGA/gelatin 70/30 material. (a) SEM image before and (b) after contact with water; (c) chemical map of the surface before and (d) after 15 days of degradation; (e) FT-IR spectra of the sample before and (f) after 15 days of degradation; (g) FT-IR spectrum of pure gelatin; (h) FT-IR spectrum of pure PLGA; (i) correlation map with respect to gelatin spectrum material; (j) deconvolution of amide I band of bioartificial material.

In a preliminary study nonmicrostructured PLGA/gelatin systems at different composition were undergone to morphological analysis before and after degradation in water. SEM images of the surfaces of a representative sample PLGA/gelatin 70/30 before and after 15 days of degradation were reported in Figures 1(a) and 1(b).

It is possible to observe a substantial change; from an initial dense morphology the sample shows a homogeneous and diffuse porosity with interconnected pores having a medium diameter of 50–100 *μ*m. The formation of these pores can be reasonably attributed to the dissolution of gelatin component that, leaving the materials, forms into the structure cavities before being filled by protein inclusions. Analogous results were obtained for samples at different PLGA/gelatin composition (data not shown); the presence of pores into scaffold structure can be considered an important aspect to favor cell colonization.

FTIR Chemical Imaging analysis confirms the presence of inclusions in the PGLA/gel structure. A representative chemical analysis of PLGA/gelatin 70/30 is reported in Figure 1. Chemical map, FT-IR spectra of sample, and correlation map with respect to gelatin spectrum (Figures 1(c), 1(e), and 1(i)) highlight a clear phase separation between synthetic and biological component; the gelatin seems to form distinct globular inclusions inside polymeric matrix, as evident in the 3D correlation map (Figure 1(i)). In particular, in Figure 1(e) the spectra acquired in two distinct points of the surface pointed out the presence of a different chemical composition; in fact the characteristic adsorption features of gelatin (amide I at 1664 cm^−1^ and amide II at 1542 cm^−1^) appeared in only one spectral region while in the other one only the adsorption peaks of PLGA are evident. On the contrary, the chemical map after degradation at 15 days (Figure 1(d)) shows a surface completely devoid of protein component, as pointed out from the absence of gelatin adsorption peaks in the spectra (Figure 1(f)) acquired in different regions, confirming that 15 days after contact with water the gelatin is completely removed from the sample. Figures 1(g) and 1(h) show pure gelatin and PLGA spectra, respectively.

In Figure 1(j), the second derivative spectra acquired from the chemical map of PLGA/gelatin 70/30, at level of gelatin inclusions, is reported. Deconvolution of amide I band allowed to identify a modification in the gelatin conformation, showing the presence of peaks that can be associated to *β*-turn conformation (1667 cm^−1^), *α* helix (1651 cm^−1^), and unordered forms (1627 cm^−1^) [38]. As already reported, FT-IR spectrum of a pure gelatin material showed an elevated variability of bands relative to amide I deconvolution (1670-1665 cm^−1^ for *β*-turn structures and 1635–1620 cm^−1^ for unordered segments). Particularly important is the effect of PLGA on gelatin that consists of an increased percentage of *α* helix structures at level of the bioartificial material.

**Figure 2:**
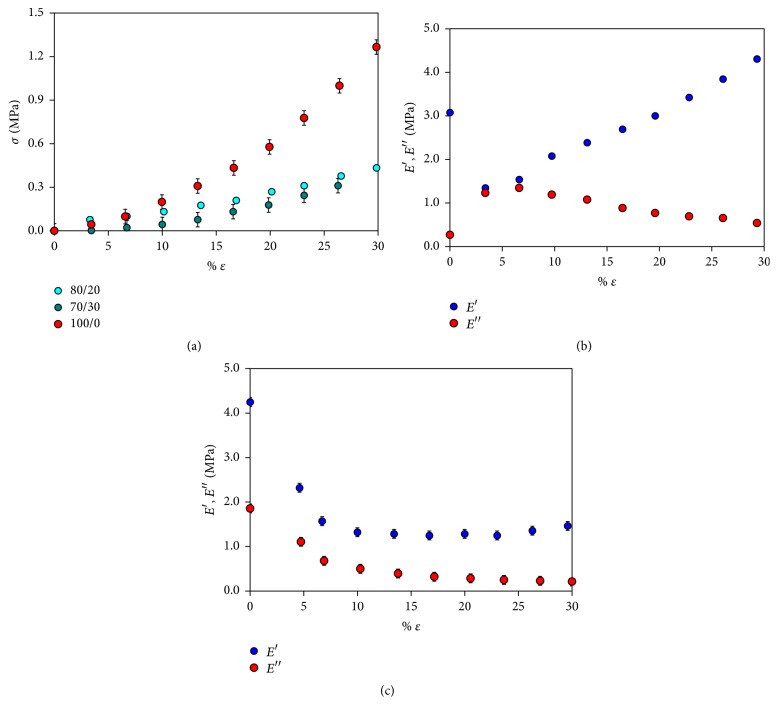
DMA analysis of nonmicrostructured PLGA/gelatin materials. (a) Curve stress-strain for PLGA pure and PLGA/gelatin 70/30 and 80/20 materials; (b) storage and loss modulus as function with deformation for pure PLGA sample; (c) storage and loss modulus as function with deformation for PLGA/gelatin 70/30 sample.

Monoaxial tests were carried out under strain control, applying a variable displacement from 0.001 mm/min to a total deformation of 30% with respect to initial length of sample. All PLGA/gelatin systems analyzed reach an imposed total deformation of 30% without fracture (Figure 2).

Bioartificial materials show an elastic modulus (*E*′ = 0.78–1.20 MPa) lower than pure PLGA material (*E*′ = 2.50 MPa) and more compatible with final application (Figure 2(a)). Dynamic tests using a cyclic deformation program were carried out by applying ten strain values in the range 0.1%–30% at a frequency of 1 Hz and for 2 min; the medium average of dynamic moduli was evaluated. The storage modulus of pure PLGA is in the range 0.13 MPa–4.00 MPa (Figure 2(b)), while PLGA/gelatin 70/30 presents a lower stiffness with storage modulus in the range of 0.23–1.42 MPa (Figure 2(c)).

**Figure 3:**
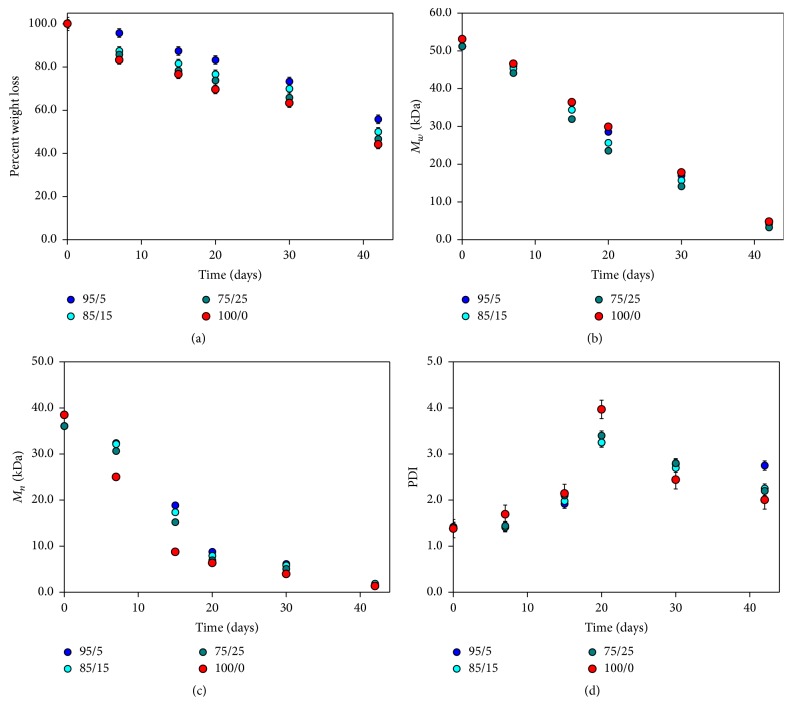
Degradation study for nonmicrostructured PLGA/gelatin materials at different composition in aqueous solution. (a) Trend of percentage weight loss versus time for PLGA/gelatin samples; (b) change of weight average molecular weight (*M*_*w*_) as function with time; (c) trend of number average molecular weight (*M*_*n*_) versus time; (d) polydispersity index (PDI) as function with time.

A more detailed analysis was carried out on bioartificial materials during the first period of degradation in aqueous solution. A pH decrease indicative of acid by-products formation is evident. The pH decrease is rather evident for pure PLGA due to its rapid degradation kinetics leading to an appreciable amount of by-products during test time. The weight loss of bioartificial materials was evaluated versus time (Figure 3(a)).

The results showed a rather rapid degradation kinetics for pure PLGA reaching a weight loss of about 55% after 42 days while bioartificial systems presented a quicker kinetics due to the presence of gelatin that is released rapidly from the systems. The bioartificial systems showed a higher degradation kinetics in the first 15 days while afterwards followed the same trend of pure synthetic polymer with values directly proportional to gelatin amount of bioartificial system. The results of GPC analysis regarding weight average molecular weight (*M*_*w*_) confirmed the effect of gelatin content on degradation kinetics (Figure 3(b)). As gelatin amount increases, the degradation kinetics is more rapid. This can be attributed to the fact that, with the increase of gelatin content, a larger number of gelatin globular aggregates are dispersed into PLGA matrix. The subsequent dissolution of protein aggregates upon aqueous incubation makes these systems more porous and then able to absorb an increased water amount with consequent increase of degradation rate. The values of numeral average molecular weight (*M*_*n*_) decrease significantly during the 20 days of analysis while successively this reduction appears less pronounced (Figure 3(c)). Regarding the results of polydispersity index (PDI) trend with time, the values show an evident increase between 7 and 20 days of test until returning to values slightly higher than those initials in the following days (Figure 3(d)).

On the basis of these results it can be hypothesized a degradation in bulk, mainly up to 20 days where a decrease of* M*_*w*_ and weight loss measurements are registered. In addition also PDI values show an increase up to 20 days indicating an important* M*_*n*_ decrease. Subsequently, a sharp* M*_*w*_ loss and PDI decrease are suggestive of a surface degradation mechanism.

#### 3.1.2. Microstructured PLGA/Gelatin Mono- and Bilayer Scaffolds

**Figure 4:**
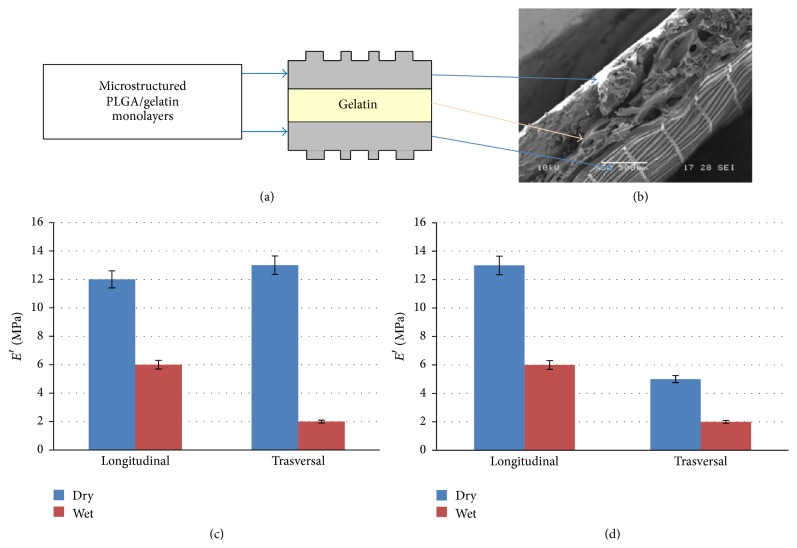
Schematic drawing (a) and SEM image (b) of a section of microstructured PLGA/gelatin 70/30 bilayer; mechanical behavior of microstructured PLGA/gelatin 70/30 monolayer (c) and bilayer (d) scaffold.

A schematic drawing and a SEM image of the section of the system PLGA/gelatin 70/30 in the form of bilayer, obtained by deposition of two microfabricated monolayers, were reported in Figures 4(a) and 4(b).

The external surface micropatterning presents well defined rectangular cell profiles and thick lines that replicate with a high accuracy the original mould. Mechanical tests were performed on the samples using strain scan method both at dry state and after immersion in a water bath. The tests were conducted by setting the elongation in both the longitudinal and transverse directions taking into account the anisotropic structure of the samples. In Figures 4(c) and 4(d), the values of* E*′ for the microfabricated PLGA/gelatin monolayer and bilayer systems are shown.

The mechanical behavior reflects significantly the anisotropy of the structure. The values of* E*′ obtained from tests carried out in the longitudinal direction, with respect to the micropatterning, were higher than those measured in the transverse direction. This difference was registered both at dry state and after immersion in water, suggesting that the anisotropic behavior can be maintained also in in vivo physiological conditions. Furthermore, the values of elastic modulus in wet condition are lower than the values measured at dry state, showing a greater compatibility with mechanical characteristics of the myocardial tissue in terms of stiffness. Both mono- and bilayer systems show mechanical characteristics in line with those of the native myocardium in terms of anisotropy and* E*′ values, particularly in wet condition where the sample stiffness tends to become more comparable to that of human myocardium measured at the end of diastole (0.2–0.5 MPa) rendering the scaffolds suitable for cardiac application [33].

**Figure 5:**
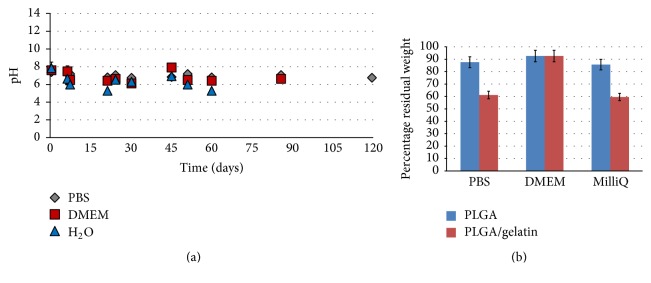
pH measurements in three different means of incubation (PBS, DMEM, and MilliQ water) for the sample microstructured PLGA/gel 70/30 at various incubation times up to 120 days (a); percentage residual weight for a microstructured PLGA/gel 70/30 sample and a microstructured PLGA pure sample, measured in PBS, DMEM, and MilliQ water after 7 days (b).

The microstructured PLGA/gelatin 70/30 systems, both single layer and bilayer, were subjected to degradation analysis in three different media: MilliQ water, PBS, and culture medium (DMEM). The analysis was carried out for a period of 6 months and at fixed times; evaluations of incubation medium in terms of pH, weight loss measurements, and morphological analysis of samples subjected to degradation were carried out. In Figure 5(a), pH values measured at various times of degradation in three different incubation media are shown.

The measurements were repeated at least three times for each sample at various times of analysis; in water the pH measurements were completed at 60 days since the material had almost completely lost its original structure. As it can be seen from the figure a significant and rapid reduction in pH occurs after degradation in water, while the values of pH measured in the buffered solutions (PBS or DMEM) decrease more moderately; this result confirms the results obtained for nonmicrostructured materials. In Figure 5(b) the results of weight loss analysis for the sample PLGA/gelatin after 7 days of incubation in three different media are reported. The values obtained for the bioartificial system were compared with those of pure PLGA system. It is possible to note how in water and PBS the loss in weight of the pure PLGA is higher compared to that registered in the case of the bioartificial system; on the contrary no difference was detected between pure PLGA and PLGA/gel after DMEM incubation, providing evidence that the release of gelatin is strongly reduced in the culture medium.

Therefore, a higher stability of the system PLGA/gelatin in culture medium can be observed, a condition more similar to that physiological, compared with the other two media, with lower percentage values of weight loss and pH values never below 6 even after complete degradation.

**Figure 6:**
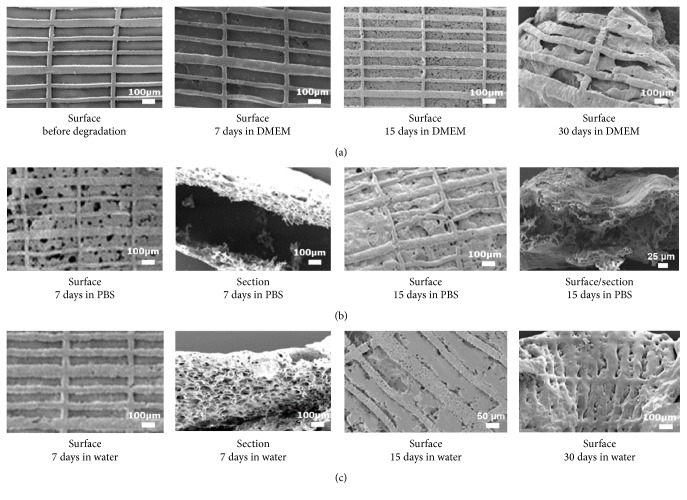
(a) SEM images of the surface of bilayer PLGA/gel 70/30, from left to right, before degradation, after 7,  15 and 30 days of incubation in DMEM; (b) SEM images of a bilayer PLGA/gel 70/30 from left to right in surface and section after 7 days, in surface after 15 and 30 days in PBS; (c) SEM images of a bilayer PLGA/gel 70/30 from left to right in surface and section after 7 days, in surface after 15 and 30 days in MilliQ water.

Comparing the SEM images of the system after contact with the culture medium (Figure 6(a)) at various degradation times, it can be observed that after 7 days the surface structure is almost unchanged and after 15 days some porosities were present, although the overall surface structure of the biomatrix is well preserved. At 30 days a significant change of the structure which appears deformed is evident. It is important, however, to note the maintenance of micropatterning, without appreciable changes in the relief and cavity dimensions. After 30 days, the matrix undergoes a collapse of the structure but, importantly, tends to preserve the imprinting of the original geometry.

In Figure 6(b), SEM images of samples of PLGA/gelatin after degradation in PBS are reported; a more rapid erosion of the matrix after contact with PBS compared to with DMEM is evident. In the first image, referring to the sample after 7 days in the presence of PBS, some porosities of the surface are already evident while in DMEM similar porosities began to appear after 15 days of incubation. The structure undergoes a collapse after 15 days and at 30 days the images show the loss of the structure which is replaced by fragments of the matrix itself. Finally, from the images obtained for PLGA/gelatin system after contact with bidistilled water (Figure 6(c)), it is possible to confirm more rapid degradation kinetics than the same biomatrices in contact with the culture medium. If at 7 days the structure is still intact, after 15 days SEM image shows a structure in which the two layers seem to interpenetrate although the micropatterning is still evident. Finally, at 30 days, the matrix is deformed and the profiles of the lines are much more irregular.

### 3.2. Stem Cell Growth and Alignment on PLGA/Gelatin Myocardial Scaffolds

hMSCs employed in this work were tested positive for CD105, CD44, CD29, CD90, and CD166 and negative for CD34, CD45, and HLA-DR, confirming their purity; their differentiation potential into both osteocyte and adipocyte lineages was also confirmed (data not shown). hMSCs were cultured on microstructured PLGA/gelatin scaffolds, and cell morphology and viability were monitored up to 15 days culture using Calcein-AM staining.

Preliminary studies of biocompatibility, carried out on scaffolds with different PLGA/gelatin ratios, showed a positive stem cell behavior (data not shown). However, the PLGA/gelatin 70/30 composition was selected for its best mechanical behavior after in vitro stretch testing.

**Figure 7:**
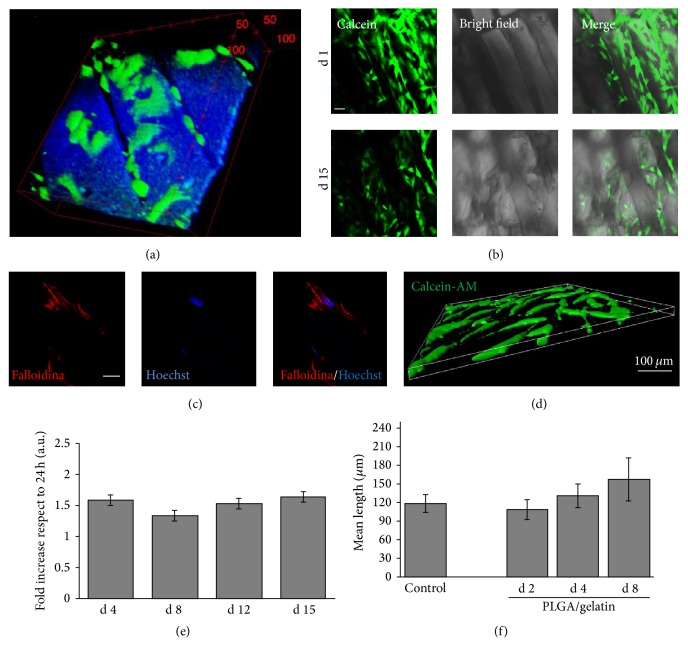
Stem cell colonization, growth, and elongation on monolayer and bilayer microstructured PLGA/gelatin scaffolds. (a) Stem cells stained with Calcein-AM and imaged using TPEF microscopy (green) visualized on a monolayer microstructured PLGA/gelatin scaffold imaged using CARS microscopy (blue) after 8 hours from seeding. Red arrows indicate a few hMSCs aligned in parallel inside the scaffold lanes. (b) Representative confocal microscopy images of living hMSCs cultured for either 1 or 15 days on bilayer scaffolds. Superposition of Calcein-AM (green) and bright field microscopy is shown. Magnification 20x, scale bar 50 *μ*m. (c) Representative confocal microscopy image showing cytoskeletal organization of hMSCs cultured for 15 days on a bilayer scaffold. Actin filaments are stained with phalloidin in red, cytoplasm with Cell Mask Green, and nuclei with Hoechst in blue. Magnification 63x, scale bar 20 *μ*m. (d) 3D reconstruction of a representative image showing hMSCs cultured for 24 h on a monolayer scaffold. Cells are stained with Calcein-AM. Magnification 20x, scale bar 100 *μ*m. (e) Cell proliferation rates after 4, 8, 12, and 15 days of culture on bilayer scaffolds. Values were normalized on cell adhesion quantified at 24 hr. (f) Mean cell lengths of hMSCs cultured on monolayer and bilayer scaffolds. Elongation measurements were performed on living cells stained with Calcein-AM at 2, 4, and 8 days of culture. Lengths were not statistically different from the 2D control.

The biocompatibility experiments were then performed on both monolayer and bilayer scaffolds, based on PLGA/gelatin 70/30 ratio, obtaining comparable results, and for this reason the results are documented through representative images of the two types of scaffolds. In the first few hours from cell seeding some of the cells began to stretch and to align in parallel to each other inside the scaffold lanes. This behavior was evident from the CARS/TPEF 3D reconstructions where the cells are shown in green, while the scaffold is imaged in blue (Figure 7(a) for one representative reconstruction).

Afterwards and within the first 24 hours, all cells adopted a stretched morphology and aligned in parallel to each other in a similar way to cell organization in native myocardium (Figure 7(b)). This morphological conformation was maintained until day 15 (Figure 7(b)) along with a preserved cytoskeletal organization (Figure 7(c)). 3D reconstructions documented the ability of living cells to colonize scaffold thickness already at an early time (24 h) (Figure 7(d)).

To quantify hMSC adhesion and growth on the scaffolds, CellTiter-Blue assay was performed. A high percentage of adhesion was registered on both monolayer and bilayer microstructured scaffolds (43.2%  ±  10.7 and 43.3%  ±  5.3, resp.). Proliferation analysis demonstrated that cells were able to proliferate on PLGA/gelatin scaffolds within day 4, with a fold increase of 1.58 ± 0.08, after which cells did not show any further significant growth on neither types of scaffolds (Figure 7(e) and data not shown). Despite proliferation arrest, cells appeared to maintain an optimal viability for the whole culture time (Figure 7(b)) and specific analysis of cell elongation confirmed that hMSCs reached an elongation comparable to 2D control cultures within day 2 and then appeared to stretch out further by day 8 (Figure 7(f)) though not in a statistically significant way. These results suggest that hMSCs cultured on microstructured PLGA/gelatin scaffolds achieve a good adhesion and scaffold colonization that is maintained for the whole culture time.

### 3.3. Cardioinductivity of PLGA/Gelatin Myocardial Scaffolds

**Figure 8:**
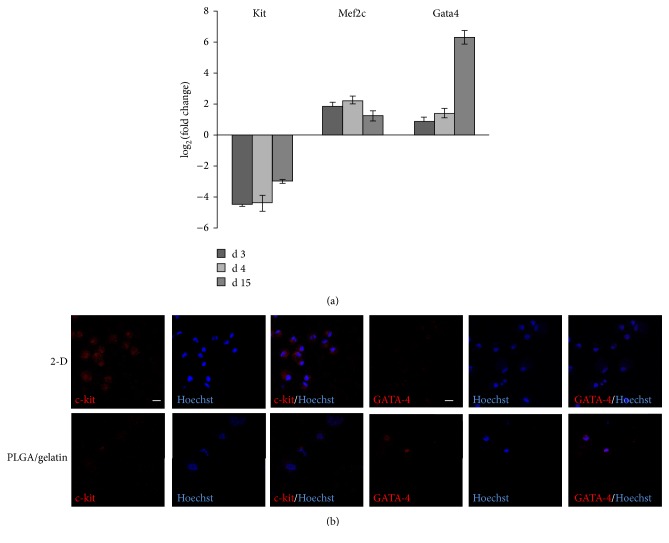
Analysis of stemness and cardiac differentiation markers. (a) qPCR results for hMSCs cultured for 3, 4, and 15 days on monolayer microstructured PLGA/gelatin scaffolds. The expression of the same genes in 2D control cultures was used for normalization. (b) Representative confocal immunofluorescence images of c-kit and GATA-4 proteins (red) and nuclei (blue) in hMSCs after either 2D control culture (top panel) or 15-day culture (bottom panel) on microstructured bilayer PLGA/gelatin scaffolds. Magnification 63x, scale bar 20 *μ*m.

To evaluate whether the culture on the microstructured scaffolds influenced gene expression in hMSCs, qPCR was performed for selected genes known to be involved in either stemness (Kit) or early cardiomyogenesis (Gata4, Mef2c) and their expression was evaluated compared to 2D control cultures. Expression of stemness marker Kit was strongly downmodulated already on day 2 in hMSCs cultured on monolayer microstructured scaffolds (−ΔΔCt corresponding to −4.47 ± 0.09). On the other hand, among the early cardiac specific transcription factors, Mef2c was the former to be upmodulated (−ΔΔCt corresponding to 1.88  ±  0.26 at 2 days) while increased transcript level of Gata4, whose expression was undetectable in 2D control cultures, began to be clearly detected after 15 days (−ΔΔCt corresponding to 6.33 ± 0.43) (Figure 8(a)).

Then, we evaluated if the increase in mRNA transcript levels was significant enough to drive to specific tissue lineage protein expression. As shown in Figure 8(b), after 15 days culture on microstructured scaffolds hMSCs showed decreased expression of the stemness marker c-kit if compared with the same cells in 2D cultures, while expression of the early cardiac transcription factor GATA-4 was acquired, confirming qPCR results. However, the static culture on these scaffolds was not associated to striated organization of cardiac contractile proteins, thereby excluding the induction of a complete sarcomeric rearrangement in these cells.

Altogether, these results show the ability of PLGA/gelatin microstructured scaffolds to direct initial hMSC lineage specification towards cardiomyogenesis, already at very early times and in the absence of any external stimuli.

## 4. Discussion

We fabricated microstructured bioartificial polymeric constructs made of PLGA/gelatin mimicking anisotropic structure and mechanical properties of the myocardium. PLGA/gelatin scaffolds promoted adhesion, ordered disposition and early myocardial commitment of hMSCs suggesting that these constructs alone, without any external stimuli, and are able to crosstalk with stem cells in a precise and controlled manner. Our in vitro degradation tests showed a favorable and controlled biomaterial degradation kinetics that suggests the use of PLGA/gelatin constructs in restoration of viable myocardium. As reported in the literature, PLGA scaffold can absorb regional ventricular wall stress to prevent infarcted myocardium before PLGA degradation [27].

In this paper we show that hMSCs cultured on microstructured PLGA/gelatin scaffolds exhibit a proliferation arrest at early times (within 4 days) and that this associates with the beginning of a cardiomyogenic differentiation process. It is important to underline that to obtain a differentiation, although at an initial level, of hMSCs in cardiac phenotype, in the complete absence of biochemical or electromechanical stimuli and thanks only to stimuli induced by intrinsic morphological and physicochemical properties of the scaffold, remains an important challenge and an innovative aspect. This behavior had been already observed for the system PHBHV/gelatin [33], at variance, we showed previously that hMSCs cultured on PHBHV/gelatin scaffolds carrying a similar microstructure proliferated for longer times and began their differentiation process only after 15 days [33]. There are several possibilities to explain this different cell behavior between PHBHV and PLGA-based scaffolds including molecular interactions between polymeric components, mechanical properties, and mainly degradation mechanism. The observed difference may be due to the polymeric composition of the scaffolds. The first key element is the molecular interaction between the synthetic polymer and gelatin which causes a conformational change of the biological component moving from random coil to a collagen-like structure [33, 39, 40]. The study of the amide I band deconvolution by FTIR Chemical Imaging shows the bands corresponding to the *α* helix, triple helix, and *β* sheet structures. However, in the case of the system based on PLGA a prevalence of bands related to *α* helix structures with respect to *β* sheet was observed. These specific conformational changes of protein component, induced by molecular interaction with PLGA, at the interface with cells could lead to a differential expression of ECM proteins, thus possibly stimulating an earlier stem cell differentiation process [41]. In addition the bands move towards lower frequencies than those observed for the system PHBHV/gelatin indicating a greater capacity of the PLGA-based system to form hydrogen bonds and to increase the material overall hydrophilicity decreasing the interfacial surface energy of the constructs. A tailored modification of surface energy and chemistry of construct can elicit the exposition of specific binding sites able to modulate the differentiation process [42].

Another consideration is that the molecular interaction occurring into bioartificial systems improves mechanical behavior with respect to pure synthetic polymer as indicated by the reduction of stiffness. Even if the direct effects of matrix stiffness to commit stem cells to a specific lineage have yet to be evaluated, many studies have demonstrated that the mechanical properties of patch materials are fundamental in supporting cell differentiation and spreading [11]. The elasticity of a cardiac patch should match that of native ventricular walls [43]. However, scaffolds based on natural polymers possess too low Young's modulus, while pure synthetic scaffolds, exhibiting higher stiffness, can induce the dedifferentiation of cells [26, 44]. An interesting study refers to the possible effect of a series of PCL substrates having the same chemistry but variable stiffness (1–133 MPa) on cardiomyocyte behavior. PCL scaffolds with Young's modulus (0.91–1.53 MPa) were found to induce mature cardiomyocytes with enhanced electromechanical coupling, while stiffer PCL scaffolds with 49.67 MPa induced the expression of immature cardiac genes like Nkx-2.5 [45]. We here proposed a bioartificial scaffold with complex physicochemical and structural properties at the mesoscale leading to stress-strain curves significantly different from those that can be obtained for dense and chemically homogenous samples [46]. Moreover, microstructured and not PLGA/gelatin scaffolds show a lower storage modulus and strain superior at 30% stress, if compared to PHBHV/gelatin scaffolds, which is even closer to the native cardiac tissue [47]. Additionally the PLGA/gelatin constructs maintain anisotropic mechanical properties already shown for PHBHV/gelatin scaffolds with a higher storage modulus value in the longitudinal direction than along the transversal direction, reflecting the myocardium anatomic anisotropy. Stem cells seeded on PLGA/gelatin scaffolds may thus receive important mechanical stimuli that will contribute to their earlier cardiomyogenic differentiation.

Lastly, stem cell behavior can be strongly influenced by the scaffold degradation kinetics. Both microstructured and not PLGA/gelatin scaffolds show a quick release of gelatin from the matrix forming a porosity suitable for a more rapid colonization of stem cells after the same cells had received the initial cardioinductive stimulus. This stimulus is given by the synergic molecular interactions and the microstructure that is maintained even when the process of erosion and dissolution is already advanced as evidenced by GPC and SEM results. Another aspect to note is that, although the overall degradation of the material is accelerated by the presence of the gelatin, from the point of view of the pH values, the presence of gelatin slows down the pH decrease avoiding the fact that a process of acidosis can be activated thus compromising cell behavior. The mechanisms by which stem cells respond to inherent material characteristics are highly complex and multicomponent. It is likely that the presence of degradation by-products in an early phase can contribute to trigger specific signals in the stem cell environment. Even if the degradation products contain acidic functionalities, some authors [48] observed that they are able to induce reduced mitosis and differentiation of smooth muscle cells. Moreover, exposure of carboxyl and hydroxyl groups derived from PLGA ester hydrolysis can activate binding of cell adhesion protein that in turn may increase cell adhesion. Nontoxic concentrations of degradation by-products led to a decrease in cell proliferation and rapid cell differentiation of human osteoblasts, and although this effect resulted detrimental for orthopedic applications, for our systems and application it confirms the possible effects of degradation products in triggering the differentiation process [49]. In addition, the manufacturing of the bioartificial system in the microstructured form makes the scaffold (mono- or bilayer) more stable during the degradation process in terms of both pH and surface topography, particularly after contact with DMEM with respect to water or PBS. This result may be considered positive taking into account that the culture medium is an environment closer to the real one. Moreover, the early cardiogenic commitment of hMSCs is an advantage especially considering the rapid degradation of the PLGA-based system so as the fusion of transplanted patch with native myocardium can take place in a shorter time for a rapid regeneration of new tissue.

Here we also show that hMSCs seeded on PLGA/gelatin scaffolds enhance Mef2c gene expression at a very early culture time (2 days), this expression is maintained at later times (3–15 days), and it is associated to an important increase of Gata4 gene expression. Mef2c is an essential transcription factor required in very early cardiac differentiation phases but it also plays a basic role in later differentiation steps by conditioning Gata4 expression. Indeed, cardiac precursors lacking Mef2c maintain Gata4 expression only in early differentiation phases; then cells lose Gata4 and this loss prevents functional cardiomyocyte formation [50, 51]. Moreover, Gata4 in turn recruits Mef2c that is a Gata4 coactivator in cardiac muscle cells and together they cooperate to activate many other cardiac promoters [52, 53]. Our present data fit previous findings and support both the strict correlation between these two genes and the positive regulatory effect of Mef2c on Gata4.

## 5. Conclusions

Microstructured PLGA/gelatin scaffolds in the form of mono- or bilayer mimicking anisotropic structure and mechanical properties of the myocardium were produced and were found to promote adhesion, long-term viability, and ordered disposition of hMSCs. hMSCs cultured on the PLGA/gelatin scaffolds exhibited a proliferation arrest at early times and this is associated with the beginning of a cardiomyogenic differentiation process. In vitro degradation tests showed a favorable and controlled biomaterial degradation kinetics that suggests the use of PLGA/gelatin constructs in restoration of a viable myocardium.

## Figures and Tables

**Table 1:** Primers used for qPCR analysis.

Gene (protein)	Forward primer sequences	Reverse primer sequences	qPCR efficiency	Length of amplicon
*Homo sapiens*				
Kit (c-kit)	5′-CTTCCTTATGATCACAAATGGGAGTT-3′	5′-TCCAGCACCCAGGGTTTTC-3′	98%	65 nt
Mef2c (MEF2C)	5′-CCATCTGCCCTCAGTCAGTTG-3′	5′-AGGCAGGGAGAGATTTGAACTCT-3′	100%	72 nt
Gata4 (GATA-4)	5′-AGCCTGGCCTGTCATCTCACTA-3′	5′-GGCCAGACATCGCACTGACT-3′	100%	73 nt
Polr2b (POLR2B)	5′-CCTGATCATAACCAGTCCCCTAGA-3′	5′-GTAAACTCCCATAGCCTGCTTACC-3′	96%	69 nt

## References

[1] Leor J., Landa N., Cohen S. (2006). Renovation of the injured heart with myocardial tissue engineering. *Expert Review of Cardiovascular Therapy*.

[2] Jana S., Tefft B. J., Spoon D. B., Simari R. D. (2014). Scaffolds for tissue engineering of cardiac valves. *Acta Biomaterialia*.

[3] Kim D.-H., Lipke E. A., Kim P. (2010). Nanoscale cues regulate the structure and function of macroscopic cardiac tissue constructs. *Proceedings of the National Academy of Sciences of the United States of America*.

[4] Stevens M. M., George J. H. (2005). Exploring and engineering the cell surface interface. *Science*.

[5] Park H., Cannizzaro C., Vunjak-Novakovic G., Langer R., Vacanti C. A., Farokhzad O. C. (2007). Nanofabrication and microfabrication of functional materials for tissue engineering. *Tissue Engineering*.

[6] Bursac N., Parker K. K., Iravanian S., Tung L. (2002). Cardiomyocyte cultures with controlled macroscopic anisotropy: a model for functional electrophysiological studies of cardiac muscle. *Circulation Research*.

[7] Gopalan S. M., Flaim C., Bhatia S. N. (2003). Anisotropic stretch-induced hypertrophy in neonatal ventricular myocytes micropatterned on deformable elastomers. *Biotechnology and Bioengineering*.

[8] Ishii O., Shin M., Sueda T., Vacanti J. P. (2005). In vitro tissue engineering of a cardiac graft using a degradable scaffold with an extracellular matrix-like topography. *Journal of Thoracic and Cardiovascular Surgery*.

[9] Engelmayr G. C., Cheng M., Bettinger C. J., Borenstein J. T., Langer R., Freed L. E. (2008). Accordion-like honeycombs for tissue engineering of cardiac anisotropy. *Nature Materials*.

[10] Jung S., Panchalingam K. M., Wuerth R. D., Rosenberg L., Behie L. A. (2012). Large-scale production of human mesenchymal stem cells for clinical applications. *Biotechnology and Applied Biochemistry*.

[11] Engler A. J., Sen S., Sweeney H. L., Discher D. E. (2006). Matrix elasticity directs stem cell lineage specification. *Cell*.

[12] Yim E. K. F., Pang S. W., Leong K. W. (2007). Synthetic nanostructures inducing differentiation of human mesenchymal stem cells into neuronal lineage. *Experimental Cell Research*.

[13] Kurpinski K., Chu J., Hashi C., Li S. (2006). Anisotropic mechanosensing by mesenchymal stem cells. *Proceedings of the National Academy of Sciences of the United States of America*.

[14] Tay C. Y., Yu H., Pal M. (2010). Micropatterned matrix directs differentiation of human mesenchymal stem cells towards myocardial lineage. *Experimental Cell Research*.

[15] McBeath R., Pirone D. M., Nelson C. M., Bhadriraju K., Chen C. S. (2004). Cell shape, cytoskeletal tension, and RhoA regulate stem cell lineage commitment. *Developmental Cell*.

[16] Faisant N., Akiki J., Siepmann F., Benoit J. P., Siepmann J. (2006). Effects of the type of release medium on drug release from PLGA-based microparticles: experiment and theory. *International Journal of Pharmaceutics*.

[17] Xin X., Hussain M., Mao J. J. (2007). Continuing differentiation of human mesenchymal stem cells and induced chondrogenic and osteogenic lineages in electrospun PLGA nanofiber scaffold. *Biomaterials*.

[18] Agrawal C. M., Ray R. B. (2001). Biodegradable polymeric scaffolds for musculoskeletal tissue engineering. *Journal of Biomedical Materials Research*.

[19] Athanasiou K. A., Niederauer G. G., Agrawal C. M. (1996). Sterilization, toxicity, biocompatibility and clinical applications of polylactic acid/polyglycolic acid copolymers. *Biomaterials*.

[20] Li W.-J., Laurencin C. T., Caterson E. J., Tuan R. S., Ko F. K. (2002). Electrospun nanofibrous structure: a novel scaffold for tissue engineering. *Journal of Biomedical Materials Research*.

[21] Telemeco T. A., Ayres C., Bowlin G. L. (2005). Regulation of cellular infiltration into tissue engineering scaffolds composed of submicron diameter fibrils produced by electrospinning. *Acta Biomaterialia*.

[22] Kim T. G., Park T. G. (2006). Biomimicking extracellular matrix: cell adhesive RGD peptide modified electrospun poly(D,L-lactic-co-glycolic acid) nanofiber mesh. *Tissue Engineering*.

[23] Li B., Li F., Ma L. (2014). Poly(lactide-co-glycolide)/fibrin gel construct as a 3D model to evaluate gene therapy of cartilage in vivo. *Molecular Pharmaceutics*.

[24] Kim K.-I., Park S., Im G.-I. (2014). Osteogenic differentiation and angiogenesis with cocultured adipose-derived stromal cells and bone marrow stromal cells. *Biomaterials*.

[25] Thevenot P. T., Nair A. M., Shen J., Lotfi P., Ko C.-Y., Tang L. (2010). The effect of incorporation of SDF-1*α* into PLGA scaffolds on stem cell recruitment and the inflammatory response. *Biomaterials*.

[26] Zong X., Bien H., Chung C.-Y. (2005). Electrospun fine-textured scaffolds for heart tissue constructs. *Biomaterials*.

[27] Zhou Q., Zhou J.-Y., Zheng Z., Zhang H., Hu S.-S. (2010). A novel vascularized patch enhances cell survival and modifies ventricular remodeling in a rat myocardial infarction model. *Journal of Thoracic and Cardiovascular Surgery*.

[28] Sarig U., MacHluf M. (2011). Engineering cell platforms for myocardial regeneration. *Expert Opinion on Biological Therapy*.

[29] Li R.-K., Yau T. M., Weisel R. D. (2000). Construction of a bioengineered cardiac graft. *Journal of Thoracic and Cardiovascular Surgery*.

[30] McCain M. L., Agarwal A., Nesmith H. W., Nesmith A. P., Parker K. K. (2014). Micromolded gelatin hydrogels for extended culture of engineered cardiac tissues. *Biomaterials*.

[31] Yacoub M. H., Terrovitis J. (2013). CADUCEUS, SCIPIO, ALCADIA: cell therapy trials using cardiac-derived cells for patients with post myocardial infarction LV dysfunction, still evolving. *Global Cardiology Science and Practice*.

[32] Navaei A., Saini H., Christenson W., Sullivan R. T., Ros R., Nikkhah M. (2016). Gold nanorod-incorporated gelatin-based conductive hydrogels for engineering cardiac tissue constructs. *Acta Biomaterialia*.

[33] Cristallini C., Rocchietti E. C., Accomasso L. (2014). The effect of bioartificial constructs that mimic myocardial structure and biomechanical properties on stem cell commitment towards cardiac lineage. *Biomaterials*.

[34] Rosellini E., Vozzi G., Barbani N., Giusti P., Cristallini C. (2010). Three-dimensional microfabricated scaffolds with cardiac extracellular matrix-like architecture. *International Journal of Artificial Organs*.

[35] Minieri V., Saviozzi S., Gambarotta G. (2015). Persistent DNA damage-induced premature senescence alters the functional features of human bone marrow mesenchymal stem cells. *Journal of Cellular and Molecular Medicine*.

[36] Mortati L., Divieto C., Sassi M. P. (2012). CARS and SHG microscopy to follow collagen production in living human corneal fibroblasts and mesenchymal stem cells in fibrin hydrogel 3D cultures. *Journal of Raman Spectroscopy*.

[37] Schindelin J., Arganda-Carreras I., Frise E. (2012). Fiji: an open-source platform for biological-image analysis. *Nature Methods*.

[38] Segtnan V. H., Isaksson T. (2004). Temperature, sample and time dependent structural characteristics of gelatine gels studied by near infrared spectroscopy. *Food Hydrocolloids*.

[39] Petibois C., Gouspillou G., Wehbe K., Delage J.-P., Déléris G. (2006). Analysis of type i and IV collagens by FT-IR spectroscopy and imaging for a molecular investigation of skeletal muscle connective tissue. *Analytical and Bioanalytical Chemistry*.

[40] Gelse K., Pöschl E., Aigner T. (2003). Collagens—structure, function, and biosynthesis. *Advanced Drug Delivery Reviews*.

[41] Chang C.-W., Hwang Y., Brafman D., Hagan T., Phung C., Varghese S. (2013). Engineering cell-material interfaces for long-term expansion of human pluripotent stem cells. *Biomaterials*.

[42] Murphy W. L., McDevitt T. C., Engler A. J. (2014). Materials as stem cell regulators. *Nature Materials*.

[43] Lakshmanan R., Krishnan U. M., Sethuraman S. (2012). Living cardiac patch: the elixir for cardiac regeneration. *Expert Opinion on Biological Therapy*.

[44] Park H., Radisic M., Lim J. O., Chang B. H., Vunjak-Novakovic G. (2005). A novel composite scaffold for cardiac tissue engineering. *In Vitro Cellular and Developmental Biology—Animal*.

[45] Forte G., Pagliari S., Ebara M. (2012). Substrate stiffness modulates gene expression and phenotype in neonatal cardiomyocytes in vitro. *Tissue Engineering Part A*.

[46] Cheng L., Sun X., Li B. (2013). Electrospun Ginsenoside Rg3/poly(lactic-co-glycolic acid) fibers coated with hyaluronic acid for repairing and inhibiting hypertrophic scars. *Journal of Materials Chemistry B*.

[47] Chen Q.-Z., Harding S. E., Ali N. N., Lyon A. R., Boccaccini A. R. (2008). Biomaterials in cardiac tissue engineering: ten years of research survey. *Materials Science and Engineering R: Reports*.

[48] Higgins S. P., Solan A. K., Niklason L. E. (2003). Effects of polyglycolic acid on porcine smooth muscle cell growth and differentiation. *Journal of Biomedical Materials Research Part A*.

[49] Meyer F., Wardale J., Best S., Cameron R., Rushton N., Brooks R. (2012). Effects of lactic acid and glycolic acid on human osteoblasts: a way to understand PLGA involvement in PLGA/calcium phosphate composite failure. *Journal of Orthopaedic Research*.

[50] Hinits Y., Pan L., Walker C., Dowd J., Moens C. B., Hughes S. M. (2012). Zebrafish Mef2ca and Mef2cb are essential for both first and second heart field cardiomyocyte differentiation. *Developmental Biology*.

[51] Karamboulas C., Dakubo G. D., Liu J. (2006). Disruption of MEF2 activity in cardiomyoblasts inhibits cardiomyogenesis. *Journal of Cell Science*.

[52] Morin S., Charron F., Robitaille L., Nemer M. (2000). GATA-dependent recruitment of MEF2 proteins to target promoters. *The EMBO Journal*.

[53] Dodou E., Verzi M. P., Anderson J. P., Xu S.-M., Black B. L. (2004). Mef2c is a direct transcriptional target of ISL1 and GATA factors in the anterior heart field during mouse embryonic development. *Development*.

